# Capturing Joint Attention: Theoretical Foundations and Their Implications for the Link Between Joint Attention and Vocabulary Growth

**DOI:** 10.1111/infa.70088

**Published:** 2026-05-09

**Authors:** Jennifer Sander, Melis Çetinçelik, Yayun Zhang, Caroline F. Rowland, Zara Harmon

**Affiliations:** ^1^ Max Planck Institute for Psycholinguistics Nijmegen the Netherlands; ^2^ Max Planck School of Cognition Leipzig Germany; ^3^ Department of Cognitive Neuroscience Faculty of Psychology and Neuroscience Maastricht University Maastricht the Netherlands; ^4^ Donders Institute for Brain, Cognition and Behaviour Radboud University Nijmegen the Netherlands

**Keywords:** joint attention, language acquisition, vocabulary acquisition

## Abstract

Despite decades of research, we still know less than we would like about the association between joint attention (JA) and language acquisition. One reason for this is that we still have not agreed how to define, operationalize, and measure joint attention. The goal of this study is to examine the impact of applying two different joint attention operationalization schemes—reflecting distinct theoretical perspectives—to the same dataset of video‐recordings of semi‐naturalistic toy‐play interactions between 12‐month‐old children and their caregivers (*N* = 39). We identified joint attention around relevant naming events to determine how these choices affect interpretations of the role of joint attention in vocabulary acquisition. We compared a gaze‐based coding scheme, consistent with associative accounts of joint attention, with a socially coordinated joint attention coding scheme, based on social‐pragmatic theories that require, in addition to gaze overlap, evidence of shared awareness. We then extracted two measures from each scheme: *average joint attention event duration* and the temporal overlap between joint attention events and naming events (*JA overlap*). We found that while measures of joint attention were predictive of later expressive vocabulary above and beyond language‐based measures in both coding schemes, model comparison based on AIC/BIC indicated that joint attention defined as coordinated JA was preferred over joint attention defined as gaze overlap. Furthermore, the best fitting model predicting later vocabulary favored predictors based on the coordinated JA scheme. Our results suggest that a social operationalization of joint attention leads to better predictors of later vocabulary size than a gaze‐based operationalization of joint attention. In addition, the current study emphasizes the critical role of methodological choices in understanding how and why joint attention is associated with vocabulary size.

## 1 Introduction

Joint attention (JA) refers to coordinated attention on a particular object or event between two interaction partners (Gabouer and Bortfeld 2021; Gale and Schick 2009; Tomasello and Farrar 1986), for example, a child‐caregiver dyad. Past research has indicated that joint attention facilitates infants' and children's word learning, and that the quality and quantity of joint attention episodes positively correlate with children's later vocabulary development (Abney et al. 2020; Adamson et al. 2004; Brooks and Meltzoff 2005; Deák et al. 2013; Morales et al. 2000; Tomasello and Todd 1983; Yu et al. 2019) and have been linked to the emergence of social cognition (Mundy and Newell 2007; Mundy et al. 2007). Joint attention is considered to provide ideal word learning conditions, as it allows for easier mapping of a word to the correct referent (Scofield and Behrend 2011).

However, despite decades of studying the role of joint attention in language acquisition, we still know less than we would like about *why* joint attention might facilitate word learning. One reason for this lack of clarity is that we still have not agreed on how to define and operationalize joint attention (Tasker and Schmidt 2008; Carpenter and Call 2013), despite the existence of a number of coding schemes (e.g., the ESCS, Mundy et al. 2003). Different conceptualisations of what is needed for a dyad to establish and maintain joint attention lead to different operationalisations, and thus to different coding schemes used to code joint attention episodes. Studies vary in their underlying definitions of what it means to achieve joint attention, which contributes to mixed findings about how and what part of joint attention facilitates language acquisition.

An example of such an inconsistent finding is the disagreement of when joint attention emerges. When joint attention is defined simply as eye contact or gaze following (e.g., Grossmann and Johnson 2010; Morales et al. 1998; Striano and Bertin 2005), it has been documented in infants as young as 5 months. Grossmann and Johnson (2010) for example used NIRS to detect brain activity in 5‐month‐old infants in a video‐based gaze following task with and without prior eye contact and found differences in the activation patterns in the prefrontal cortex of these children. Children's brains were sensitive to the social cue of eye contact, indicating early sensitivity to joint attention in triadic interactions already present at 5 months of age. However, this study tested the ability of the children's brains to distinguish between an interaction with or without joint attention, operationalized as eye contact, but not the level of children's behavioral responsiveness or own engagement in joint attentional episodes. When joint attention is defined by the child needing to actively engage in more complex coordinated joint interactions themselves, then joint attention has been found to emerge late in the first or early in the second year of life (e.g., Carpenter et al. 1998; Tomasello 1995; Bakeman and Adamson 1984; see Mundy et al. 2007 for similar evidence that different dimensions of JA (e.g., directing the attention of others vs. responding to JA requests from others) have different relationships with vocabulary acquisition). Disagreements in underlying concepts of joint attention, and thus in the way that data are acquired, coded, and measured lead to difficulties in identifying why joint attention facilitates word learning and which aspects of joint attention might be most strongly related to word learning.

The aim of this paper is to examine how definitional vagueness in the construct of joint attention affects the interpretation of empirical findings. We aim to evaluate how operationalisations of joint attention map onto different theoretical positions, and to test whether these operationalisations make different empirical predictions in our data. Using the same dataset allows us to avoid drawing conclusions based on population differences—a potential contributor to mixed findings of previous studies—and instead to compare the influence of the coding schemes on the conclusions we can draw.

There are, at least, two ways to operationalize joint attention, and its relationship with language, linked to two different theoretical perspectives. On the one hand we have associative theorists (e.g., Samuelson and Smith 1998; Corkum and Moore 1998) reasoning that low‐level shared visual attention underlies the mechanism by which joint attention facilitates word learning. On this view, the combination of endogenous sustained and object‐focused shared visual attention is sufficient for joint attention to facilitate word learning. Following this view, the relationship between joint attention and word learning is due to co‐occurrence of attention between a label and the corresponding object or action. Overlap in attention helps the child narrow down the possible referents to the intended one over time and map a label to its corresponding referent. Defining joint attention by the co‐occurrence of attention to labels and objects leads to studies that operationalize joint attention in their coding schemes in terms of shared visual attention onto the same object at the same time by both interaction partners. These schemes use measures such as the amount of time that dyads spend looking, simultaneously, at the same referent during naming events to quantify joint attention. We call this a *gaze overlap scheme* (see, e.g., Abney et al. 2017; Yu et al. 2019).

On the other hand, we have social pragmatic accounts claiming that an additional active social awareness component of joint attention is required to facilitate word learning (Akhtar et al. 1996; Tomasello 1995; MacGowan et al. 2021). On this view, joint attention operationalisations start from the premise that shared visual attention in the form of gaze overlap is not enough to define a joint attentional episode. Instead, attentional overlap needs to be accompanied by social awareness of this shared attention, evidenced by, for example, a verifying look toward the dyad partner (Tomasello and Farrar 1986; Gabouer and Bortfeld 2021). For shared attention to be facilitative of language, social pragmatic accounts require social awareness of the shared attention and the accompanying social‐pragmatic cues involved in joint attention. The child needs to understand the caregiver's intention of connecting labels and objects for this interaction to support the child's word learning (Tomasello 1995). This intention reading allows children to infer the meaning of labels they hear over time. Thus, on this view, one must, in addition to shared (visual) attention, in some way code a shared social awareness that indexes active coordination of attention on the part of at least one member of the dyad. Defining joint attention by the presence of social awareness leads to studies that operationalize joint attention in their coding schemes in terms of shared attention in combination with an indicator of social awareness (e.g., intentionality indicated by, for example, mutual gaze between the interaction partners). We call this a *coordinated joint attention (coordinated JA)* scheme (see e.g., Gabouer and Bortfeld 2021; Tomasello and Farrar 1986).

To compare the two coding schemes, we tested how well they predicted children's expressive vocabulary size at 15 and 18 months. Our analyses focused on six predictors: two baseline predictors measuring child language that were independent of the coding schemes and two predictors measuring joint attention properties that were dependent on the coding schemes, applied to each coding scheme separately.

The baseline predictors were the *expressive vocabulary size at 12 months* and the *number of naming events per minute* per dyad during a semi‐naturalistic toy play session when children were 12 months old. Both measures were chosen as they represent approximations of language knowledge and language input at the timepoint of behavioral testing, which have been shown to be reliable predictors of later language abilities (Feldman et al. 2005; Mahr and Edwards 2018). Including these as a baseline allows us to assess the effect of both joint attention coding schemes beyond basic language abilities and language input.

The two coding scheme‐specific predictors were the *JA overlap* per dyad and the *average JA event duration* per dyad. These were coded in two different ways, once for all joint attention events identified by the *gaze overlap scheme* and once for all joint attention events identified by the *coordinated JA scheme*. Depending on the underlying joint attention definition, a variety of different measures can be deducted from a joint attention episode, but not all joint attention definitions allow for the deduction of the same measures. The measures we chose represent a frequency measure (of *JA overlap*) and a duration measure of the joint attention event (*average JA event duration*), assessing the most fundamental characteristics of a joint attention event. A more detailed explanation follows in the Method section below.

This procedure allowed us to determine whether applying the two coding schemes described above to exactly the same dataset would lead us to draw different conclusions about the relationship between joint attention and language acquisition.

## 2 Method

### 2.1 Participants

This study formed part of a longitudinal project (the Language 0–5 Project; Rowland et al. 2018). The present study was conducted according to the guidelines laid down in the Declaration of Helsinki, with written informed consent obtained from a parent or guardian for each child before any assessment or data collection. All procedures involving human subjects in this study were approved by the University of Liverpool Research Ethics Committee. Ninety‐five monolingual British English‐speaking families were recruited: an initial sample of 89 families when the child was 6 months of age and an additional six families at 15 months of age. One child was excluded because of a persistent ear infection, and four families did not continue after the initial visit. By the end of data collection (at age 4;6), a further 13 had dropped out. All infants were born at full term, none were of low birth weight, and all were typically developing when recruited. All dyads were from Merseyside, United Kingdom. The majority of the caregivers identified themselves and their child as White (88 White, 1 Mixed ethnicity: White and Other, 1 Other ethnic group). Socioeconomic status was overall high: Maternal education was high (0 no formal qualifications, 6 GCSE level or similar, 10 A level or similar, 34 University degree or similar, 40 Postgraduate or similar), Paternal education was high (2 no formal qualifications, 13 GCSE level or similar, 16 A level or similar, 42 University degree or similar, 17 Postgraduate or similar) and annual household income was high (0 £0–£14,000, 3 £14,001–£24,000, 24 £24,001–£42,000, 62 £42,001 or more, 1 declined to answer, categories were based on the UK tax brackets at the time of data collection).

For this study, participants were mother‐child dyads who took part in a semi‐naturalistic toy play session in the lab when their children were 12 months old. Dyads were excluded if, for either of the interaction partners, gaze data was detectable from the video‐recordings of the session for less than 20% of the time (*n* = 41). Participants were also excluded if they did not have at least one (successful) joint attention event identified based on each coding scheme (*n* = 6), as necessary measures could otherwise not be computed, or if vocabulary data were missing (*n* = 2). Dyads were also excluded if the caregiver was a father (*n* = 1), as the majority of participating caregivers were mothers. After exclusion, 39 dyads remained in the dataset. Children were 12 months old at the time of semi‐naturalistic data collection and the first vocabulary datapoint (mean age = 12; 09 months, SD = 7.44 days, range: 11; 28–12; 26) and 15‐month (mean age = 15; 11 months, SD = 7.13 days, range = 15; 0–15; 25) and 18‐month (mean age = 18; 18 months, SD = 7.01 days, range = 18; 04–19; 03) old at the time we measured their vocabulary again. The sample size of the original dataset was determined based on power analyses for the analyses for the planned statistical tests. For the present study, the sample size was dictated by the data available, as this study represents a secondary data analysis.

### 2.2 Measures and Procedure

#### 2.2.1 Vocabulary Checklist

Children's concurrent and subsequent receptive and expressive vocabulary size was assessed at 12, 15, and 18 months of age through a parental report checklist, the British English CDI (Alcock 2020), which is an adaption of the Mac‐Arthur‐Bates Communicative Development Inventory (CDI, Fenson et al. 2006). Out of a list of 396 items, caregivers ticked off the words their children knew (receptive vocabulary) and the words their children knew and said (expressive vocabulary). For the current study, we used the 12‐month and 15‐month expressive CDI scores to parallel previous studies investigating effects of joint attention on later vocabulary development that have used 3‐month time intervals between behavioral measures and vocabulary measures (e.g., Yu et al. 2019), and because the 15‐month data showed the highest coefficient of variation (12‐month = 1.17, 15‐month = 1.37, 18‐month = 0.91). However, results with 18‐month data can be found in the supplementary material and preliminary results with both 15‐ and 18‐month data can also be found in Sander et al. (2024). The 15‐month expressive CDI score was our dependent measure (*expressive vocabulary size at 15 months*) and the 12‐month expressive CDI score was a dyad‐specific predictor (*expressive vocabulary size at 12 months*). For our dependent variable, *expressive vocabulary size at 15 months* (and at *18 months*, see supplementary materials), we calculated the number of known and produced items as well as the number of not known and not produced items.

#### 2.2.2 Semi‐Naturalistic Data

##### 2.2.2.1 Experimental Setup

Children and their caregiver engaged in a semi‐naturalistic toy play situation with one of two possible sets of toys in a lab for about 10 min. Each set consisted of a stacking toy (rings or cups), a ball, a rubber duck, a teething ring (bear or giraffe), a rainmaker or a ragdoll and a play phone, and a variety of different rattles (a flower or a dog; a giraffe rattle and turtle rattle or one with green and purple ends). Interactions were audio‐video‐recorded using two cameras, providing different angles; one from a tripod‐fixated digital camera and one hand‐held by the researcher. Interaction between the dyad and the researcher was kept to a minimum. Caregivers were instructed to interact with the child as they normally would.

##### 2.2.2.2 Data Processing

The semi‐naturalistic data were pre‐processed and annotated in the video analysis program ELAN (Version 6.4, Max Planck Institute for Psycholinguistics Nijmegen, The Language Archive, 2022) and post‐processed and analyzed in *R* (R Core Team 2023; Wickham 2016). The data, as well as the code used for processing and analysis are available at: https://osf.io/vtg9x. In ELAN, audio‐video data from the hand‐held camera were annotated for caregivers' speech (categorized into naming events, description of objects, comments on objects, incomprehensible, and other non‐relevant speech), and both interaction partners' gaze location (onto relevant objects, their co‐participant, and other non‐relevant locations), throughout the whole recording. Then, around naming events, the data were annotated for attentional behaviors (pointing, waving, etc.), and touch (to the object or co‐participant). These annotations were completed prior to the application of the two joint attention coding schemes. Intercoder reliability for these annotations was, on average, 90% (ranging from 70% agreement for parental gaze to 99% agreement for parental naming events).

#### 2.2.3 Joint Attention Coding

For both coding schemes, we focussed on joint attention around naming events, which are events for which the synchronization of children's attention on the object and its naming by the caregivers is crucial for word learning (Schroer and Yu 2022a, 2022b). Naming events were all utterances of the caregiver labeling a present object (e.g., ‘This is a duck!’).

##### 2.2.3.1 Gaze Overlap Scheme

The *gaze overlap scheme* was based on that of Yu et al. (2019). Joint attention was defined as a period of shared and continuously aligned visual attention of both interaction partners onto an object of interest for a minimum of 500 ms, allowing for short looks away by the caregiver for a maximum of 300 ms. We analyzed joint attention events around naming events that overlapped for at least 1 ms with naming events.

##### 2.2.3.2 Coordinated Joint Attention Scheme

The *coordinated JA scheme* was based on the coding scheme developed by Gabouer and Bortfeld (2021). The coding scheme is based on previous work on coordinated joint attention stemming from a social pragmatic perspective and has been developed as a general joint attention coding scheme applicable to various interaction types. We coded each naming event for the presence of: (1) an initiation attempt, which can consist of a number of behaviors (e.g., relevant speech, touch, or other attentional behaviors addressed at the object or interaction partner) performed with the intention to share attention with the interaction partner, followed by (2) a response from the interaction partner, which can likewise consist of a number of relevant behaviors, followed by (3) a validation behavior in which the joint attention initiator has to validate the interaction partner's response by again showing some behavior addressed at the interaction partner or object. To be coded as a *successful* joint attention event, the three behaviors had to be intentional and not accidental. Here, intention was assessed through indicators such as visual attention, physical orientation, haptic interaction, or overt gestures toward the object of interest (Trueswell et al. 2016; Gabouer and Bortfeld 2021). This allowed us to differentiate between events that were intentional to share attention and those in which one interaction partner, for example, accidently knocked over an object (touch, but not intentional), or an event in which an interaction partner dug through all the toys searching for a specific other toy. Moreover, the behaviors had to occur in the right order (initiation‐response‐validation) within a specific timeframe (e.g., after each step, the onset of the following step had to occur within 5 seconds), and the period of shared attention had to last at least 3 seconds. Joint attention ended when one interaction partner disengaged with both the interaction partner and the target object for a period of 5 seconds or longer (Gabouer and Bortfeld 2021). This disengagement period also represents the minimal time window for identifying a joint attention initiation: a behavior can be considered as a joint attention behavior only if no successful joint engagement with the corresponding object has been observed in the 5 seconds before the considered joint attention initiation behavior.

##### 2.2.3.3 Comparison of the Two Joint Attention Coding Schemes

**FIGURE 1:**
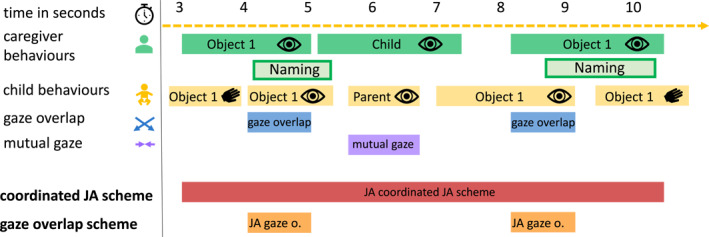
Schematic overview of behavior and joint attention annotations. Behavior annotations (e.g., gaze—represented by the eye symbols, touch—represented by the hand symbols, naming events) for caregiver (green) and child (yellow) in a 10‐s sample from the original video data. Overlapping gaze on the object (blue) and mutual gaze (violet) are identified. Joint attention as identified by the *gaze overlap scheme* is shown in orange and occurs if there is gaze overlap for at least 500 ms. This results in two instances in the schematic overview: first, between the onset and offset of gaze overlap on Object 1 between second 4 and 5, and second, between the onset and offset of gaze overlap on Object 1 between second 8 and 9. Joint attention as identified by the *coordinated JA scheme* is visualized in red, and occurs if both interaction partners engage in joint attentional behaviors (including gaze, touch, and other attentional behaviors) in a specific temporal relationship that indicates social awareness of both interaction partners within the ongoing shared attentional episode and all minimal duration requirements are fulfilled.

The *gaze overlap scheme* and the *coordinated JA scheme* differ not only in the underlying definition of joint attention, but also in their operationalization of joint attention. Importantly, the differences in these coding schemes lead to different segments of the interaction being identified as joint attention, which then have different characteristics (see Figure 1). e.g., in our data, the average joint attention event as defined by the *gaze overlap scheme* lasted 2.99 s, while the average joint attention event as defined by the *coordinated JA scheme* lasted 28.97 s. Different coding schemes thus identified different joint attention events with different characteristics.

#### 2.2.4 Measures Derived From the Semi‐Naturalistic Data

##### 2.2.4.1 Number of Naming Events Per Minute

For each dyad, we calculated the number of naming events the caregiver uttered per minute during the available playtime for annotation. All naming events, including repetitions of already uttered naming events within the session, were considered. Naming events of other things or objects not present in the room as well as non‐naming‐event related utterances and utterances between the caregiver and experimenter were not considered. This measure represents an approximation of the language input rate for each dyad during the toy play session, intended as a coarse approximation of the everyday rate of language input for that dyad.

We measured joint attention in two ways: *JA overlap* and *JA event duration*. For each dyad, and for each joint attention event identified by each coding scheme, we applied both of these measures.

#### 2.2.5 JA Overlap

This measure was adapted from Yu et al. (2019). We calculated the mean proportion of overlap between (successful) joint attention events and naming events for each dyad, which provides a measure of joint attention i.e. independent of the number of naming events.

#### 2.2.6 Average JA Event Duration

For each dyad, we calculated the average duration of all joint attention events that were identified around naming events.

In total, we derived seven measures for each dyad, two derived from the vocabulary checklist and five from the semi‐naturalistic data.

1. concurrent *expressive vocabulary size at 12 months*, the time point of the behavioral assessment, per dyad
2. the later *expressive vocabulary size at 15 months*
3. the *number of naming events per minute* per dyad as a language input measure
4. the *JA overlap* per dyad based on joint attention events identified by
  - The gaze overlap scheme
  - The coordinated JA scheme
5. *average JA event duration* per dyad based on joint attention events identified by
  - The gaze overlap scheme
  - The coordinated JA scheme

*Number of naming events per minute*, *expressive vocabulary size at 12 months*, *JA overlap* and *average JA event duration* were log‐transformed to reduce skew prior to analysis.

### 2.3 Statistical Analysis

We investigated the relationship between joint attention and later expressive vocabulary size in three sets of analyses. In the first set of analyses, we investigated whether each of the two different joint attention coding schemes predicted later expressive vocabulary size beyond the two baseline measures, *expressive vocabulary size at 12 months* and the *number of naming events per minute*. We compared two coding scheme‐specific models with a baseline model. In the second set of analyses, we determined which coding scheme‐specific model, with its set of coding scheme‐specific measures, yielded the better fit to *expressive vocabulary size at 15 months* in a direct comparison. To do so, we compared the AIC and the BIC values of the two models and conducted a 10‐fold cross‐validation. Cross‐validation was used to assess model performance on held‐in and held‐out data, providing an estimate of generalizability to unseen data. In our final set of analyses, we determined which individual measures of joint attention derived from the two different coding schemes best predicted later vocabulary size and how these measures were related to each other. We assessed this in two steps: first, by examining a correlation matrix to evaluate relationships among the four predictors; and second, by applying a model averaging procedure to identify consistently supported patterns while taking into account model selection uncertainty.

## 3 Results

### 3.1 Naming Events

We identified a total of 634 naming events across all 39 dyads that were accompanied by sufficient gaze data, fulfilling the minimal requirement to be included in our analyses.

Because the two schemes have different criteria for joint attention, they do not identify the same joint attention events (see Table 1). Out of the 634 naming events, 116 were identified as associated with joint attention events by both coding schemes. Eighty‐four were associated with joint attention as measured by the *gaze overlap scheme* but not associated with joint attention as measured by the *coordinated JA scheme*, and 186 were associated with joint attention as measured by the *coordinated JA scheme* but not by the *gaze overlap scheme*. The remaining 270 naming events were not associated with joint attention in either coding scheme.

**TABLE 1:** Distribution of all naming events considered in both coding schemes.

		Coordinated JA scheme	
		Naming events with JA	Naming events without JA
Gaze overlap scheme	Naming events with JA	116	84
	Naming events without JA	186	248

*Note:* Naming events were either surrounded by a joint attention event or not surrounded by a joint attention event. This was assessed separately for each coding scheme, allowing for four potential combinations: naming events were considered to be surrounded by joint attention by both, none, or one of the coding schemes.

We assessed concurrent (*expressive vocabulary size at 12 months*) and subsequent (*expressive vocabulary size at 15 months*) expressive CDI scores for each child. Mean *expressive vocabulary size at 12 months* was 7.33 (SD = 8.61), and mean *expressive vocabulary size at 15 months* was 30.69 (SD = 42.08; note total possible score = 395). The mean *number of naming events per minute* was 2.07(SD = 1.56). *JA overlap* and *average JA event duration* were assessed separately for each joint attention coding scheme. The mean *JA overlap* score was 22.4% (SD = 13.96) in the *gaze overlap scheme* and 46.02% (SD = 18.87) in the *coordinated JA scheme*. The *average JA event duration* was 2.99 s (SD = 2.38) in the *gaze overlap scheme* and 28.97 s (SD = 22.96) in the *coordinated JA scheme* (See Table 2 for more details).

**TABLE 2:** Descriptive statistics for all seven predictors.

	Mean (SD) [range]	
Expressive vocabulary size at 15 months	30.69 (SD = 42.08 [0–240]) items of 395	
Expressive vocabulary size at 12 months	7.33 (SD = 8.61 [0–39]) items of 395	
Number of naming events per minute	2.07 (SD = 1.56 [0.13–8.2]) naming events per minute	

	**Gaze overlap scheme**	**Coordinated JA scheme**
JA overlap	22.4 (SD = 13.96 [4.47–58.97]) %	46.02 (SD = 18.87 [2.9–89.82]) %
Average JA event duration	2.99 (SD = 2.38 [1.04–11.7]) s	28.97 (SD = 22.96 [4.21–125.69]) s

### 3.2 Comparison Between Baseline Model and Coding Scheme Specific Models

Our first set of analyses examined the relationship between joint attention as measured by each coding scheme, and later *expressive vocabulary size at 15 months*. To assess whether JA predictors were predictive above and beyond language‐based predictors, we compared a baseline model containing only the predictors *expressive vocabulary size at 12 months* and *number of naming events per minute*, with two full models, one for each coding scheme, which contained, as well as the baseline measures, the coding scheme‐specific predictors *JA overlap* and the *average duration of JA events* as predictors of later vocabulary size. For each of the three models, we ran a binomial regression predicting the *expressive vocabulary size at 15 months* (number of total items known and used and number of total items not known and not used) from the following predictors: *number of naming events per minute* and *expressive vocabulary size at 12 months* in the baseline model and in the coding scheme‐specific models, and *JA overlap* and *average JA event duration* only in the coding scheme‐specific models. *Number of naming events per minute*, *expressive vocabulary size at 12 months*, *JA overlap* and *average JA event duration* were log‐transformed to reduce skew prior to analysis.

**FIGURE 2:**
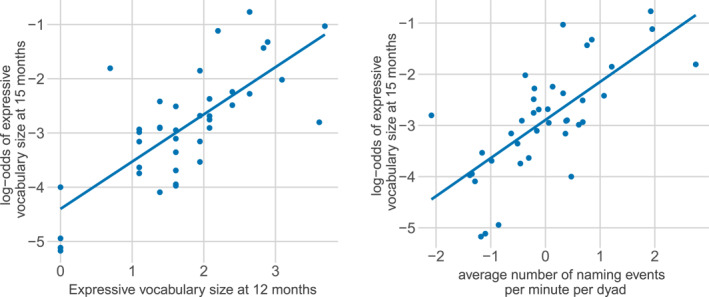
Log odds of a vocabulary item of the expressive CDI at 15 months being reported as known and used, modeled as a function of the baseline predictors. Log‐odds of a vocabulary item of the expressive CDI at 15 months being reported as known and used (*y*‐axis) modeled as a function of the observed values (*x*‐axis) for the two log‐transformed baseline predictors in the baseline model: the expressive vocabulary size at 12 months (left), the average number of naming events per minute (right) per dyad. Lines shows regression line, dots show scores for individual dyads.

Our baseline model shows that both of our baseline measures, expressive *vocabulary size at 12 months* (*β =* 0.83, *z =* 21.48, *p <* 0.001) and *number of naming events per minute* (*β =* 0.71, *z =* 22.85, *p <* 0.001) were positively associated with *expressive vocabulary size at 15 months* (Figure 2).

**FIGURE 3:**
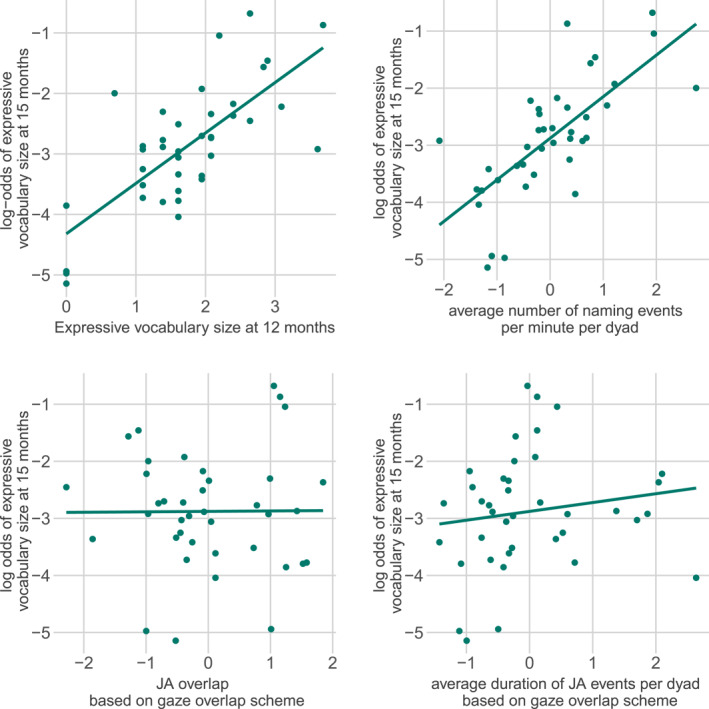
Log odds of a vocabulary item of the expressive CDI at 15 months being reported as known and used (*y*‐axis) modeled as a function of the observed values (*x*‐axis) for the log‐transformed predictors based on the *gaze overlap scheme*: the two baseline measures in the upper row: *expressive vocabulary size at 12 months* (top left), and the *average number of naming events per minute* (top right); and the two coding scheme‐specific measures on the bottom: *JA overlap* (left), and the *average JA event duration* (right) stemming from the *gaze overlap scheme*. Lines show regression line, dots show scores for individual dyads.

The significant effect of the two baseline measures on *expressive vocabulary size at 15 months* remained when we added the variables from the *gaze overlap scheme* (*expressive vocabulary size at 12 months*: *β =* 0.85, *z =* 21.38, *p <* 0.001, *number of naming events per minute*: *β =* 0.68, *z =* 20.83, *p <* 0.001). Beyond the baseline measures, there was also a significant positive effect of *JA overlap* (*β =* 0.12, *z =* 3.68, *p* < 0.001), suggesting that dyads with a higher average overlap of joint attention and naming events had a larger vocabulary size at 15 months. However, there was no significant effect of *average JA event duration* (*β =* −0.06, *z =* −1.57, *p =* 0.12, see Figure 3). Model comparison showed that the *gaze overlap scheme* model fitted the vocabulary data better than the baseline model (ΔAIC: 10.14, ΔBIC: 6.82), suggesting that the joint attention measures used in the *gaze overlap scheme* explained additional variance over and above the two baseline measures.

**FIGURE 4:**
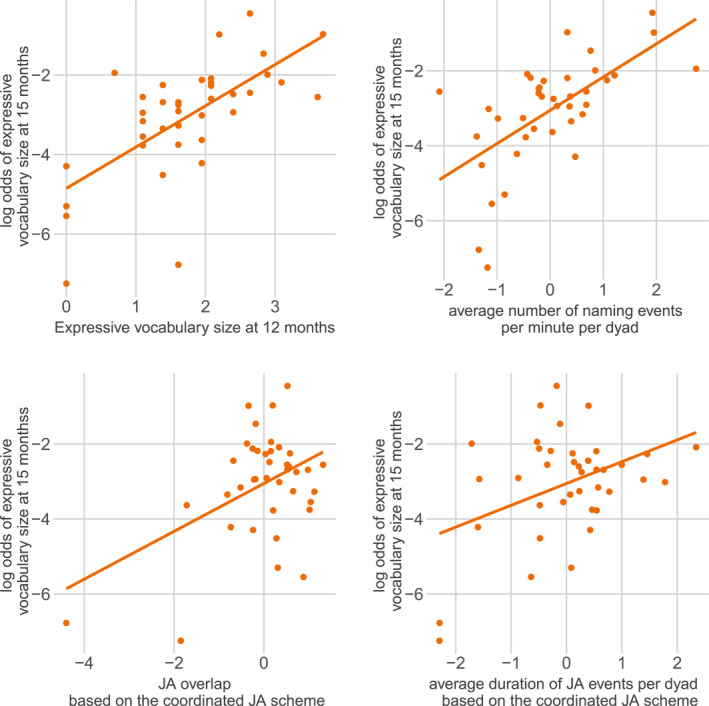
Log odds of a vocabulary item of the expressive CDI at 15 months being reported as known and used (*y*‐axis) modeled as a function of the observed values (*x*‐axis) for the log‐transformed predictors based on the *coordinated JA scheme*: the two baseline measures in the upper row: *expressive vocabulary size at 12 months* (top left), and the *average number of naming events per minute* (top right); and the two coding scheme specific measures on the bottom: *JA overlap* (left), and *average JA event duration* (right) stemming from the *coordinated JA scheme*. Lines show regression line, dots show scores for individual dyads.

The significant effect of the two baseline measures on *expressive vocabulary size at 15 months* also remained when we added the coding‐scheme specific predictors from the *coordinated JA scheme* (*expressive vocabulary size at 12 months*: *β =* 0.98, *z =* 22.67, *p <* 0.001, *number of naming events per minute*: *β =* 0.82, *z =* 24.55, *p <* 0.001). There was also a significant positive effect of *average JA duration* (*β =* 0.36, *z =* 8.56, *p <* 0.001) suggesting that dyads with longer joint attention had larger vocabulary size at 15 months. Finally, there was a significant positive effect of *JA overlap* (*β =* 0.38, *z =* 5.89, *p* < 0.001, see Figure 4), suggesting that dyads with more overlap between joint attention events and naming events had children with larger vocabulary sizes at 15 months of child age. Model comparison revealed that the *coordinated JA scheme* model fitted the vocabulary data better than the baseline model (ΔAIC: 137.81, ΔBIC: 134.48), suggesting that the joint attention measures used in the *coordinated JA scheme* explained additional variance over and above the two baseline measures.

### 3.3 Comparison of the Coding Scheme Specific Models

In this section, we use model comparison to evaluate the performance of our two joint attention models. First, we recapitulate the AIC and BIC values of both models from our previous analyses. Second, we report on the results of a 10‐fold cross‐validation for robust model performance estimation.

The *coordinated JA scheme* model yielded both a lower AIC and a lower BIC, suggesting a better fit to the vocabulary data than the *gaze overlap scheme* model. See Table 3 for the results.

**TABLE 3:** AIC and BIC values of both models, the gaze overlap scheme model and the coordinated JA scheme model.

	Gaze overlap model	Coordinated JA model	Δ
AIC	822.48	694.81	**ΔAIC: 127.67**
BIC	830.80	703.13	**ΔBIC: 127.67**

*Note:* Bold values indicate the delta, representing the difference between the two AIC/BIC values.

For the 10‐fold cross validation, the dataset was divided into 10 equal parts of which nine folds were used for training and the remaining fold was used for testing. Testing was repeated 10 times, each time with a different fold being the test set. The evaluation was based on three metrics: the mean absolute error (MAE), the root mean squared error (RMSE), and *R* ^2^. MAE and RMSE both quantify prediction error, with lower values indicating a better model fit. *R* ^2^ indicates the proportion of variance in the dependent variable that is captured by the model, with higher values indicating better model fit.

The model based on the joint attention measures from the *coordinated JA scheme* outperformed the model based on the joint attention measures of the *gaze overlap scheme*, evident by the lower mean MAE, lower mean RMSE, and higher mean *R* ^2^ (see Table 4). Based on the cross‐validation, the *coordinated JA scheme* model outperforms the *gaze overlap scheme* model.

**TABLE 4:** Results of the cross validation.

Metric	Minimum	1st quantile	Median	Mean	3rd quantile	Maximum	NA's
MAE							
Gaze overlap model	0.01	0.04	0.04	0.06	0.09	0.11	0
Coordinated JA model	0.01	0.03	0.04	0.05	0.07	0.09	0
RMSE							
Gaze overlap model	0.01	0.05	0.06	0.09	0.11	0.20	0
Coordinated JA model	0.01	0.04	0.05	0.07	0.09	0.17	0
*R* ^2^							
Gaze overlap model	0.03	0.24	0.56	0.57	0.97	0.99	0
Coordinated JA model	0.02	0.42	0.72	0.62	0.85	0.97	0

*Note:* Comparison of MAE, RMSE, and *R* ^2^ for both models, the model based on the gaze overlap scheme and the model based on the coordinated JA scheme.

### 3.4 Comparison of JA Measures

Finally, we investigated which measure based on which coding scheme best predicts later vocabulary size. In all analyses above we used two measures: a measure of overlap of JA and naming events (*JA overlap*) and a duration measure of JA (*average duration of JA events*). Our analyses above showed that these measures yielded different results when applied to different coding schemes. When operationalized according to the *gaze overlap scheme*, the *JA overlap* measure predicted vocabulary size, but the *average JA event duration* measure did not. When operationalized according to the *coordinated JA scheme*, both the *JA overlap* as well as the *average JA event duration* predicted vocabulary size.

To gain insight on how predictors are related to each other and to ensure that our models are not built with redundant information, we first assessed the correlations between our measures. We then conducted a model averaging procedure. Model averaging is useful for cases where competing models could explain the data in different ways. By averaging over multiple models rather than relying on a single ‘best’ model, model averaging procedures allow us to evaluate how different predictors perform across multiple models. This enables us to better understand the relative contribution of the coding‐scheme specific measures to predicting vocabulary.

**FIGURE 5:**
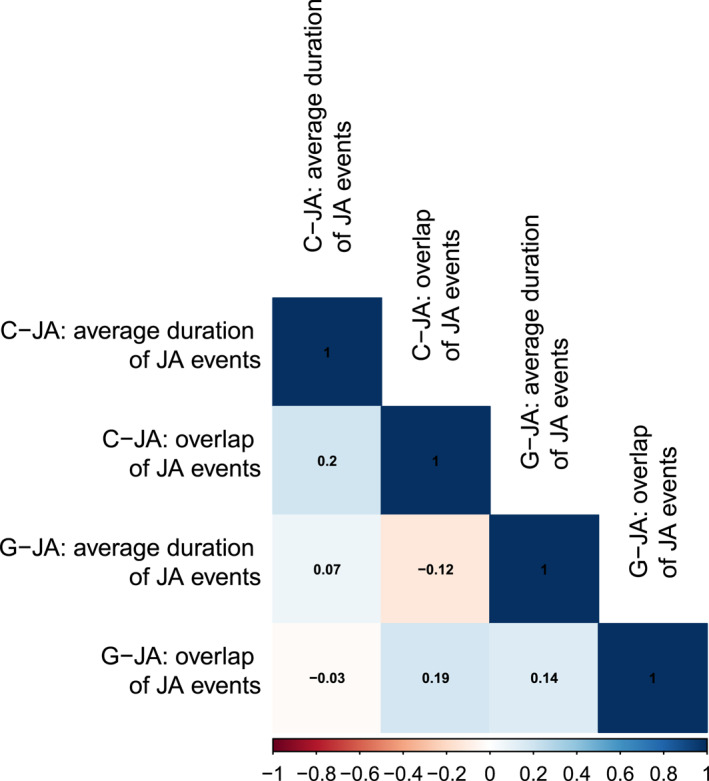
Correlation matrix of the two coding scheme‐specific measures applied to the two joint attention coding schemes. From top to bottom/left to right: *average JA event duration* (*coordinated JA scheme*), *JA overlap* (*coordinated JA scheme*), *average JA event duration* (*gaze overlap scheme*), *JA overlap* (*gaze overlap scheme*). Colors indicate strength and direction of correlation: red indicates a negative correlation and blue indicates a positive correlation. Intensity of the colors indicate the strength. The upper diagonal in dark blue contains the number +1, indicating full correlation as these represent the correlation of each predictor with itself.

The correlation matrix (see Figure 5) showed weak correlations between the *JA overlap* and *average JA event duration* measures of each coding scheme (correlation between the *JA overlap* and the *average JA event duration* in the gaze overlap scheme = 0.14; correlation between the *JA overlap* and *average JA event duration* in the coordinated JA scheme = 0.2). Further, there were weak correlations of measures across coding schemes (*JA overlap* = 0.19, *average JA event duration* = 0.07). The fact that the correlations were only weak suggests that the four measures are not redundant in the way that they capture different aspects of JA, potentially because their underlying constructs of JA differ.

Model averaging was conducted using the MuMIn package in *R*, identifying the predictors out of all available predictors that are consistently important for explaining the dependent variable. We included both baseline predictors, the *number of naming events per minute* and the concurrent *expressive vocabulary size at 12 months*, and the two coding scheme‐specific predictors from both coding schemes, *JA overlap* and *average JA event duration*. The dependent variable was again the *expressive vocabulary size at 15 months*. The full average model was identified by averaging all candidate models whose ΔAIC with the best model was below 5, accounting for model uncertainty.

The results indicated that the two baseline predictors, the *number of naming events per minute* and the concurrent *expressive vocabulary size at 12 months*, showed again a significant positive effect on the proportion of CDI items known and used at 15 months (*number of naming events per minute*: *β =* 0.82, *z = 23*.*1*, *p < 0*.*001*; expressive *vocabulary size at 12 months*: *β =* 1.01, *z =* 21.06, *p < 0*.*001*). For the coding scheme‐specific measures, the *JA overlap* measure as well as the *average JA event duration* from the *coordinated JA scheme* were again significant positive predictors of later language (*JA overlap*: *β = 0*.*38*, *z = 5*.*66*, *p < 0*.*001*; *average JA event duration*: *β = 0*.*39*, *z = 8*.*41*, *p < 0*.*001*). In line with our findings in the first set of analyses of this paper, the *average JA event duration* measure stemming from the *gaze overlap scheme* was not a predictor of later language (*average JA event duration*: *β = −0*.*1*, *z = 1*.*96*, *p = 0*.*05*). Differing from our previous findings for the *gaze overlap scheme* model, we found also no significant effect of the *JA overlap* predictor stemming from the *gaze overlap scheme* in the model averaging (*JA overlap*: *β = −0*.*00082*, *z = 0*.*05*, *p = 0*.*96*). The model‐averaged coefficients are summarized in Table 5. In sum, the both coding scheme‐specific measures from the *coordinated JA scheme* significantly predicted later language and the coding scheme‐specific measures from the *gaze overlap scheme* did not. In other words, longer and more frequent joint attention events as measured by the *coordinated JA scheme* were associated with larger vocabulary size.

**TABLE 5:** Results of the model averaging procedure.

Predictors	Expressive vocabulary at 15 months (CDI)			
	Estimates	SE	*Z*	*p*
(Intercept)	−4.81	0.11	40.66	< 0.001
Expressive vocabulary size at 12 months	1.01	0.05	21.06	< 0.001
Number of naming events per minute	0.82	0.03	23.10	< 0.001
Coordinated JA scheme: Average JA event duration	0.39	0.04	8.41	< 0.001
Coordinated JA scheme: JA overlap	0.38	0.07	5.66	< 0.001
Gaze overlap scheme: Average JA event duration	−0.1	0.05	1.96	0.05
Gaze overlap scheme: JA overlap	−0.0008	0.01	0.05	0.96

*Note*. Effect on *expressive vocabulary size at 15 months* of all potential predictors: *expressive vocabulary size at 12 months*, *number of naming events per minute*, *JA overlap* (*gaze overlap scheme*), *average JA event duration* (*gaze overlap scheme*), *JA overlap* (*coordinated JA scheme*), *average JA event duration* (*coordinated JA scheme*).

*p < 0.05. **p < 0.01. ***p < 0.001.

Note too that we conducted the same set of analyses with the expressive vocabulary scores at 18 months of child age, and results, overall, confirmed our findings for the 15 months expressive vocabulary scores (see supplementary materials, Supporting Information S1; Table S1–S4 and Supporting Information S1; Figures S1–S3).

## 4 Discussion

The goal of the current study was to examine the impact of applying two different joint attention operationalization schemes to the same dataset of video‐recordings of semi‐naturalistic toy‐play interactions between 12‐month‐old children and their caregivers (*N* = 39). In three sets of analyses we investigated the relationship between joint attention (JA) and later expressive vocabulary size. Across all three sets of analyses we found the two language‐related baseline predictors, *expressive vocabulary size at 12 months* and the *number of naming events per minute*, to be a consistent positive predictor of later *expressive vocabulary size at 15 months*.

In our first set of analyses, we investigated whether each of the two different joint attention coding schemes predicted later expressive vocabulary size beyond our two baseline measures. Both coding schemes outperformed the baseline model. However, for the *gaze overlap scheme*, only *JA overlap* was a significant positive predictor of later *expressive vocabulary size at 15 months*. The second coding scheme‐specific predictor, *average JA event duration*, was not a significant predictor. For the *coordinated JA scheme* both coding scheme‐specific predictors were significant positive predictors of later *expressive vocabulary*. Our findings suggest that both coding schemes measure relevant properties of joint attention in the way that they improve the models' ability to predict language beyond the two baseline measures, *expressive vocabulary at 12 months* and the *number of naming events per minute*. However, they differ in which coding scheme‐specific measures predict later vocabulary size.

In the second set of analyses, we determined which coding scheme model, with its set of coding scheme‐specific measures, yielded the better fit to *expressive vocabulary size at 15 months* in a direct comparison. In both the AIC and the BIC comparison, the *coordinated JA scheme* model yielded a better fit to the data. This finding was replicated using cross‐validation: the *coordinated JA scheme* model explained a larger proportion of the variance of the data than the *gaze overlap scheme* model. Overall, the results of the second set of analyses suggest that while measures of joint attention were predictive of later expressive vocabulary size above and beyond language‐based measures in both coding schemes, the model comparison based on AIC/BIC indicated that joint attention defined as coordinated JA was preferred over joint attention defined as gaze overlap. Furthermore, the best fitting model predicting later vocabulary size favored predictors based on the *coordinated JA scheme*.

In our final set of analyses, we determined which individual measures of joint attention derived from the two different coding schemes best predicted later vocabulary size and how these measures were related to each other. We assessed this in two separate steps, using a correlation matrix and a model averaging procedure. Assessing the correlations of all four measures available, we found only weak or no correlations between the measures, suggesting that they are tapping into different aspects of joint attention. In a second step, we conducted a model averaging procedure with all four coding scheme‐specific predictors in addition to the two baseline measures. The results of the model averaging suggest that both coding scheme‐specific measures, *average JA event duration* and *JA overlap*, stemming from the *coordinated JA scheme* were significant positive predictors of later expressive vocabulary size. Neither of these coding scheme‐specific measures stemming from the *gaze overlap scheme* was a predictor of *expressive vocabulary size at 15 months*. Except for the non‐significant effect of *JA overlap* stemming from the *gaze overlap scheme*, the results of the model averaging are in line with the results from our first set of analyses. The finding that *JA overlap* from the *gaze overlap scheme* is predictive in the *gaze overlap scheme* model but not in the averaged model means that *JA overlap* no longer shows a significant effect when accounting for the effects of the coding scheme‐specific measures of the *coordinated JA scheme*.

Overall, taking all analyses together, the coding scheme‐specific measures derived from the *coordinated JA scheme* consistently predict later *expressive vocabulary size at 15 months*. They also consistently outperformed the measured derived from the *gaze overlap scheme*. The finding that longer coordinated joint attention episodes with higher overlap between joint attention and naming events are associated with larger vocabularies is consistent with the idea that extended joint attention contexts coincide with richer opportunities for word–referent mapping. Joint attention episodes identified by the *gaze overlap scheme* do not suggest the same relationship, or, in regards to *JA overlap*, only weakly.

We applied two different coding schemes to the exact same dataset. Based on these, we identified two different sets of joint attention events. Applying the same measures (*JA overlap* and *average JA event duration*) to these different sets of joint attention events, we measured different joint attention properties, which suggest different relationships between joint attention and later vocabulary size. For the *coordinated JA scheme*, we find a clear and consistent relationship between joint attention and later vocabulary. For the *gaze overlap scheme* we do not find the same relationship. This finding highlights the objective of the present study: the choice of a joint attention coding scheme has significant influence on how we conceptualize the relationship between joint attention and language acquisition. Only for the *coordinated JA scheme* did we find a consistent relationship between joint attention and children's later expressive vocabulary. The subsequent analyses of the same predictors applied to 18 months expressive vocabulary scores further underline these findings: While we confirmed our findings that the coordinated JA scheme outperforms the gaze overlap scheme also for 18 months expressive vocabulary scores, we find differences in the strength of single predictors, suggesting an age sensitivity to when which predictor from which coding scheme finds a relationship to children's later expressive vocabulary.

The current analyses provide evidence about the consequences of competing operationalisations, rather than direct evidence for the theories themselves. At the same time, operationalisations are the interface between theory and data; therefore, differences in results across operationalizations highlight important tensions in the theoretical claims (see Fried 2020 for more detail on distinction between statistical and theoretical models). It is important to note that we have examined only two different theories. There are, of course, other ways to break down joint attention. For instance, Mundy et al. (2007) categorize joint attention along four dimensions and compare three theories in terms of what they predict about the development of these dimensions. However, each dimension in Mundy et al. is operationalized in only one way; that is, it is implicitly assumed that these operationalisations are equally valid across all three theories. What we have shown, however, is that this assumption does not necessarily hold: the same operationalization scheme cannot straightforwardly be used to compare theories, because each theory operationalizes joint attention in a different way. Crucially, the choices made about operationalization can have a substantial impact on the results. Thus, although we have not tested different theories from Mundy (and from other papers), we believe our general point applies to all of them: theories differ not only in what they predict, but also in how they operationalize joint attention, and this needs to be taken into account. The two coding schemes in this study measure somewhat different underlying joint attention constructs. We will now discuss reasons why this may be the case.

First, the two coding schemes differ fundamentally in their basic assumptions of the mechanism underlying joint attention and, consequently, in the way they operationalize joint attention. In case of the *gaze overlap scheme*, the combination of endogenous object‐focused shared visual attention is sufficient for joint attention to facilitate language acquisition. Accordingly, joint attention is identified by the presence of gaze overlap between the child and their caregiver. For the *coordinated JA scheme* this is not enough. Instead, joint attention requires the fulfillment of the three‐step procedure of joint attention initiation, target response, and verification. Only if all three steps are present and take place in a specific time window, it can be ensured that both interaction partners are actively aware of the shared attention. Further, it requires evidence of active coordination of attention. In the *coordinated JA scheme* this social awareness of the shared attention is operationalized by the requirement of intentionality of sharing attention, and intention reading by the co‐participant.

It has been suggested that word learning is enabled not through a passive associative process but an active process enabled through the mechanism of intention reading: in this view, children learn not through the cooccurrence of object and label but through identifying the object a label is *intended* to refer to (see Baldwin 1995 for a strong argument in support of this view). In experiments, children were able to learn labels for objects in ostensive and nonostensive contexts, as long as the intended object was identifiable (Tomasello and Barton 1994; Baldwin 1991; Baldwin et al. 1996). Shared intentionality is considered unique to human cognition and potentially a driver of human communication (Tomasello et al. 2005); for example, Mundy and Newell (2007) argue, based on developmental, neurocognitive and clinical evidence, that joint attention is the attention‐based system underlying social cognition, which allows children to understand others as intentional agents. In this view joint attention provides the mechanism for learning from social interactions (Mundy and Newell 2007). The requirement of intentionality of the *coordinated JA scheme* accounts for these findings and is in line with this view. In addition, the necessity of intentionality in the *coordinated JA scheme* separates the two coding schemes in their fundamental assumptions of how (human) children learn through joint attention. These differences in the operationalization of joint attention result in different events being classified as joint attention. In the gaze overlap scheme, any instance that meets the minimal temporal requirements is always classified as joint attention, whereas in the *coordinated JA scheme*, such instances only qualify as joint attention if combined with social awareness. As a consequence, we identified different numbers of joint attention events in the exact same dataset, depending on which coding scheme was applied (see Table 1).

Second, the coding schemes differ not only in how they define, operationalize and measure joint attention, they also differ in how they account for sustained attention. It has been suggested that sustained attention to an object or event of interest is the actual driver of vocabulary learning and that joint attention works as a facilitator of sustained attention in infants (Yu and Smith 2016; Yu et al. 2019). The study the *gaze overlap scheme* stems from measures joint attention and sustained attention separately and finds that the interaction of sustained and joint attention facilitates children's word learning abilities. Meanwhile, the framework which the *coordinated JA scheme* stems from, does not account for sustained attention separately. However, the temporal requirements of the coding scheme require dyads to maintain joint attention for a much longer period of time than in the *gaze overlap scheme*, basically enforcing sustained attention to be included in joint attentional periods within the *coordinated JA scheme*. This difference in how the coding schemes incorporate or separate sustained and joint attention could be one of the reasons for why we find joint attention to relate to vocabulary size differently depending on the applied coding scheme.

Third, the coding schemes differ in the *number and variety of behaviors* they use to operationalize joint attention. The *gaze overlap scheme* operationalizes joint attention by gaze overlap only, excluding non‐gaze related behaviors and mutual gaze between the interaction partners. In the *coordinated JA scheme*, gaze is not the only behavior that is being considered, but dyads' engagement in several possible behaviors are taken into account. In addition to gaze, the *coordinated JA scheme* also considers touch or general attentional behaviors such as pointing and tapping—behaviors that have been shown to influence a child's attention (Deák et al. 2018; Smith et al. 2023; Suarez‐Rivera et al. 2019). Touch, in particular, has been argued to serve as the primary mode of caregiver‐child interaction and may represent a foundation for the emergence of joint attention (Botero 2016). Previous research suggests that in hearing speaking dyads not gaze overlap alone, but the coupling of gaze and object touch and the alignment of attention through eye‐hand‐coordination facilitates children's word learning (Yu and Smith 2013; Schroer and Yu 2022a, 2022b). Further, the operationalization of joint attention in the *coordinated JA scheme* does not limit eye gaze to sharing gaze onto the same object, but also allows for mutual gaze, a form of gaze of crucial importance in all, but especially in interactions using a sign language (Lieberman et al. 2014). This flexibility allows a variety of behaviors to co‐occur and overlap as the interaction partners maintain or establish JA by the means of these behaviors, which is also important because it is likely that different behaviors are of different relevance at different ages. For younger children, overlapping visual attention appears particularly important, with evidence that mutual gaze in infants predicts later language development (e.g., Morales et al. 1998; Striano and Bertin 2005). With increase in age, children's behavioral repertoire changes, likely affecting their engagement in joint attention (Rohlfing and Nomikou 2014; Sander et al., under revision). This suggests that different operationalisations may capture joint attention differently at different ages.

Fourth, the schemes differ in their *temporal granularity*. In particular, they differ in the required minimal duration of joint attention (*gaze overlap scheme*: 500 ms vs. *coordinated JA scheme*: 3000 ms) and the duration of disengagement which terminates an ongoing joint attention event (*gaze overlap scheme*: 300 ms vs. *coordinated JA scheme*: 5000 ms). This results in a different time scale of JA events. The difference in duration might also explain why we found no effect for the *average JA event duration* stemming from the *gaze overlap scheme* in either of the analyses: The strict and short time limitations in the *gaze overlap scheme* might have kept their duration so stable that not enough variance was present to add explanatory power to our model. In contrast, the broader definition of the *coordinated JA scheme* requires us to consider a longer timescale, including longer periods of interruption. Allowing for longer periods of interruption is in line with the dynamics of the social situation. Even though granularity is not a consequence of the joint attention definition, our findings motivated future research into the effect of defining joint attention at different temporal granularities.

As well as the differences explored in our study, which applied to interactions with hearing‐seeing children, it is also important to note that the schemes differ in their *suitability for interactions between blind and/or deaf individuals*. Because the *gaze overlap scheme* operationalizes joint attention as shared visual attention onto an object of interest, it cannot be used with blind individuals. Whether blind individuals are considered to be able to engage in joint attention then depends on the applied definition of joint attention. Definitions and coding schemes of joint attention that require gaze overlap would have to draw the conclusion that blind individuals do not engage in joint attention. Definitions and coding schemes of joint attention that allow for different behaviors and define joint attention as coordinated joint engagement can potentially consider joint interactions of blind individuals as joint attention, for example through joint touch (for the relevance of tactile joint attention initiation in sighted children see Wang et al. 2025).

Similar issues arise when we consider applying the coding schemes to interactions between individuals using a sign language. It has been shown that signing individuals engage more often in mutual gaze and in more frequent gaze switching between the object or action of interest and their interaction partner, compared to speaking dyads (Lieberman et al. 2011, 2014). Even though shared gaze onto an object of interest is possible in signing dyads, it rarely overlaps with the timepoint of naming. Mutual gaze is required for a sign to be successfully communicated, so signing dyads spend more time in mutual gaze than speaking dyads (Lieberman et al. 2014). The *gaze overlap scheme* would reject these important instances of language exchange in signing dyads as joint attention. Thus, this scheme is likely not the best choice for investigating joint attention in the environment of interactions using a sign language. The *coordinated JA scheme*, allowing for mutual gaze, touch, and other attentional behaviors, shows a higher level of flexibility and has successfully been applied to interactions in a sign language before (e.g., American Sign Language (ASL); Sander et al. 2025). And note that, even though other behaviors beyond gaze are of special interest in some populations, in fact non‐gaze related joint attention behaviors occur in all interactions. And finally, it is necessary to point out that many of the world's children grow up in situations in which they experience relatively little joint attention, as not all societies and groups of individuals engage in joint attention in the ways observed in the present and related studies (Akhtar 2013; Akhtar and Gernsbacher 2007). More research is needed to identify the different pathways through which joint attention or other forms of interaction facilitate language learning in different social contexts and populations. Further, future work might look at extending schemes like the gaze overlap scheme to broaden their definition of what behaviors could be included in joint attention episodes.

One limitation of the current study is that joint attention was only assessed around naming events. We were interested especially in the characteristics of joint attention events surrounding naming events, as these are the instances that allow for successful mapping between words and objects. However, this could have skewed the results, as fruitful joint interaction that did not lead to a naming event was not considered. Including joint attention events not associated with a naming event in our current dataset could have changed the frequency and duration of joint attention events. As the comparison of the coding schemes was the main goal of the present study, we chose this procedure despite the limitations associated with it.

Another potential limitation of the current study concerns the inherent subjectivity embedded in the coordinated joint attention scheme. Although there are strong theoretical grounds for including intentionality as a defining criterion into the coding scheme, individuals' intentions cannot be directly or objectively measured. Consequently, any assessment of intentionality necessarily remains inferential and approximate (e.g., what looks like intentionality to one coder might not to another, what looks like intentionality to the coder may not be an intentional action by the child). That said, there was high inter‐rater reliability between our coders, which suggests that they were at least interpreting the same behaviors as intentional.

A third potential limitation concerns the low variability of expressive vocabulary scores at 12‐month, which may constrain the strength of the observed relationship with later language measures. Nonetheless, 12‐month expressive vocabulary size was a significant predictor of 15‐ and 18‐month expressive vocabulary size, suggesting that early individual differences, despite their restricted range, contained meaningful information about subsequent vocabulary growth. Finally, a large number of dyads from the original dataset had to be excluded, due to insufficient gaze data. Because the recordings were not originally designed for this study, the quality of visual data varied. This may have introduced some selection bias, as included dyads may differ in subtle ways (e.g., positioning) from those that were not included. Future research using data collected for the purpose of this study could help ensure more complete gaze information and confirm the robustness of the found patterns in a larger sample.

In sum, the findings of the present study show that how we define joint attention determines not only how we operationalize joint attention but also how we measure it, and, critically, whether we find a relation between joint attention and language development. These definitions can vary widely, from definitions that rely only on gaze overlap (i.e., the associative accounts) to ones that also include a variety of behaviors that can signal mutual awareness of the interactional partners' attention. As our results show, the choice of joint attention definition and operationalization influences which conclusions can be drawn about the relationship between joint attention and language development. We found different relationships between joint attention and vocabulary size while applying the same measures to the same dataset with the same dependent variable but based on different joint attention coding schemes.

Our results demonstrate that whether the empirical evidence supports a particular theory of the role of joint attention in development of a system (in our case vocabulary size) critically depend on how we define, operationalize and measure joint attention. This shows the critical importance of having well defined and specific theories of what we think joint attention actually is and of reporting in detail the conceptualisations, definitions and measures of joint attention we are applying to our data (Hutmacher and Franz 2025). Only then we can compare study results and draw conclusions beyond the results of single studies.

Choosing an appropriate scheme for a given participant group, making explicit decisions about the granularity of joint attention, as well as choosing the relevant measures of joint attention represent a challenging and understudied aspect of research on joint attention. Future research with careful and clear methodological choices is needed to understand which aspects of joint attention might facilitate language acquisition.

## Author Contributions

**Jennifer Sander:** conceptualization, methodology, software, data curation, investigation, validation, formal analysis, project administration, visualization, writing – original draft, writing – review and editing. **Melis Çetinçelik:** conceptualization, data curation, investigation, project administration, software, validation, writing – review and editing. **Yayun Zhang:** conceptualization, project administration, supervision, writing – review and editing. **Caroline F. Rowland:** writing – review and editing, conceptualization, funding acquisition, investigation, project administration, resources, supervision. **Zara Harmon:** writing – review and editing, validation, conceptualization, formal analysis, methodology, project administration, software, supervision.

## Ethics Statement

This study formed part of a longitudinal project (the Language 0–5 Project; Rowland et al. 2018) approved by the University of Liverpool Research Ethics Committee.

## Conflicts of Interest

The authors declare no conflicts of interest.

## Supporting information


Supporting Information S1

## Data Availability

The data that support the findings of this study are openly available in OSF at https://osf.io/vtg9x/.
