# Supplementary material for: Capturing Joint Attention: Theoretical Foundations and Their Implications for the Link Between Joint Attention and Vocabulary Growth
Source: Infancy. 2026 May 9;31:e70088. doi: 10.1111/infa.70088 (PMC13157240; doi:10.1111/infa.70088)
Supplement: Supplementary file 1 — Supporting Information S1 [file INFA-31-0-s001.docx]

**Supplementary Materials**

To examine the relationship between the predictors of the gaze overlap scheme and the coordinated joint attention scheme not only on the expressive vocabulary size at 15 months but also at 18 months, we conducted subsidiary analyses. These parallel the analyses from the manuscript: we first compared a baseline model with two full models containing the coding scheme-specific predictors. We then conducted a model comparison to evaluate the performance of the two joint attention models. We recapitulated AIC and BIC values of both models and report the results of a 10-fold cross-validation for robust model performance estimation. We finally conducted a comparison of the JA measures by conducting a model averaging procedure.

**Supplementary Table 1**

*Descriptive statistics for all seven predictors.*

|  | **Mean (SD) [Range]** | |
| --- | --- | --- |
| **Expressive vocabulary size at 18 months** | 92.82 (SD=84.48 [1-324]) items of 395 | |
| **Expressive vocabulary size at 12 months** | 7.33 (SD=8.61 [0-39]) items of 395 | |
| **Number of naming events per minute** | 2.07 (SD=1.56 [0.13-8.2]) Naming Events per minute | |
|  | **Gaze Overlap Scheme** | **Coordinated JA Scheme** |
| **JA overlap** | 22.4 (SD=13.96 [4.47-58.97]) % | 46.02 (SD=18.87 [2.9-89.82]) % |
| **Average JA event duration** | 2.99 (SD=2.38 [1.04-11.7]) sec | 28.97 (SD=22.96 [4.21-125.69]) sec |

**Comparison between baseline model and coding scheme specific models**

Our first set of analyses examined the relationship between joint attention as measured by each coding scheme, and later *expressive vocabulary size at 18 months*. To assess whether JA predictors were predictive above and beyond language-based predictors, we compared a baseline model containing only the predictors *expressive vocabulary size at 12 months* and *number of naming events per minute*, with two full models, one for each coding scheme, which contained, as well as the baseline measures, the coding scheme-specific predictors *JA overlap* and the *average duration of JA events* as predictors of later vocabulary size. For each of the three models, we ran a binomial regression predicting the *expressive vocabulary size at 18 months* (number of total items known and produced and number of total items not known and not produced) from the following predictors: *number of naming events per minute* and *expressive vocabulary size at 12 months* in the baseline model and in the coding scheme-specific models, and *JA overlap* and *average JA event duration* only in the coding scheme-specific models*.* *Number of naming events per minute*, *expressive vocabulary size at 12 months*, *JA overlap* and *average JA event duration* were log-transformed to reduce skew prior to analysis.

Our baseline model shows that both of our baseline measures, expressive *vocabulary size at 12 months* (*β =* 0.60, *z =* 25.13, *p <* .001) and *number of naming events per minute* (*β =* 0.40, *z =* 20.39, *p <* .001) were positively associated with *expressive vocabulary size at 18 months* (Supplementary Figure 1).
**Supplementary Figure 1** *Log odds of a vocabulary item of the expressive CDI at 18 months being reported as known and used, modelled as a function of the Baseline Predictors*

**
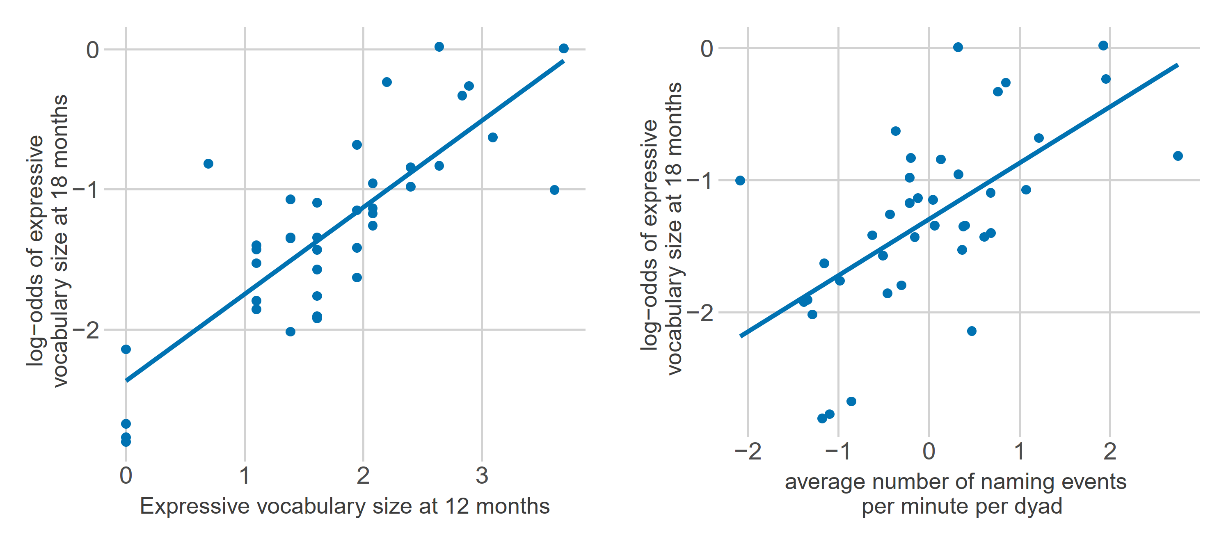
**

*Note.* Log-odds of a vocabulary item of the expressive CDI at 18 months being reported as known and used (y-axis) modelled as a function of the observed values (x-axis) for the two log-transformed baseline predictors in the baseline model: the *expressive vocabulary size at 12 months* (left), the *average number of naming events per minute* (right) per dyad. Lines shows regression line, dots show scores for individual dyads.

The significant effect of the two baseline measures on *expressive vocabulary size at 18 months* remained when we added the variables from the *gaze overlap scheme* (*expressive vocabulary size at 12 months*: *β =* 0.69, *z =* 26.22, *p <* .001, *number of naming events per minute*: *β =* 0.37, *z =* 18.13, *p <* .001). Beyond the baseline measures, there was also a significant positive effect of *JA overlap* (*β =* 0.12, *z = 5.61*, *p* < .001), suggesting that dyads with a higher average overlap of joint attention and naming events had a larger vocabulary size at 18 months. Finally, there was a significant negative effect of *average JA event duration* (*β =* -0.18, *z =* -7.95, *p=* < .001, see Supplementary Figure 2). Model comparison showed that the *gaze overlap scheme* model fitted the vocabulary data better than the baseline model (ΔAIC: 74.23, ΔBIC: 70.90), suggesting that the measures used in the *gaze overlap scheme* explained additional variance over and above the two baseline measures.

**Supplementary Figure 2***Log odds of a vocabulary item of the expressive CDI at 18 months being reported as known and used, modelled as a function of the Predictors of the Gaze Overlap Scheme Model*


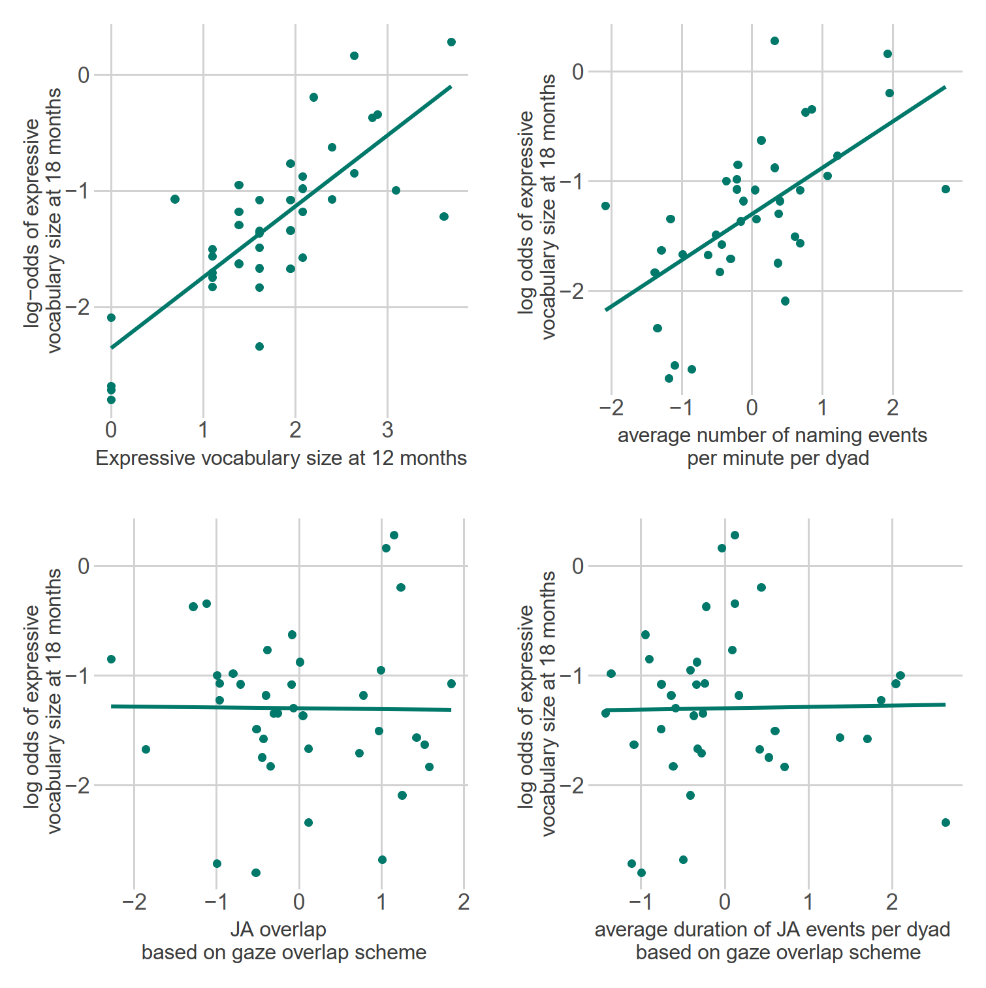

*Note.* Log odds of a vocabulary item of the expressive CDI at 18 months being reported as known and used (y-axis) modelled as a function of the observed values (x-axis) for the log-transformed predictors based on the *gaze overlap scheme*: the two baseline measures in the upper row: *expressive vocabulary size at 12 months* (top left), and the *average number of naming events per minute* (top right); and the two coding scheme-specific measures on the bottom: *JA overlap* (left), and the *average JA event duration* (right) stemming from the *gaze overlap scheme*. Lines show regression line, dots show scores for individual dyads.

The significant effect of the two baseline measures on *expressive vocabulary size at 18 months* also remained when we added the coding-scheme specific predictors from the *coordinated JA scheme* (*expressive vocabulary size at 12 months*: *β =* 0.71, *z =* 27.12, *p <* .001, *number of naming events per minute*: *β =* 0.47, *z =* 21.96, *p <* .001). There was also a significant positive effect of *average JA duration* (*β =* 0.42, *z = 17*, *p <* .001) suggesting that dyads with longer joint attention had larger vocabulary size at 18 months. However, there was no significant effect of *JA overlap* (*β =* 0.04, *z =* 1.32, *p* = .19, see Supplementary Figure 3). Model comparison revealed that the *coordinated JA scheme* model fitted the vocabulary data better than the baseline model (ΔAIC: 355.39, ΔBIC: 352.07), suggesting that the measures used in the *coordinated JA scheme* explained additional variance over and above the two baseline measures.


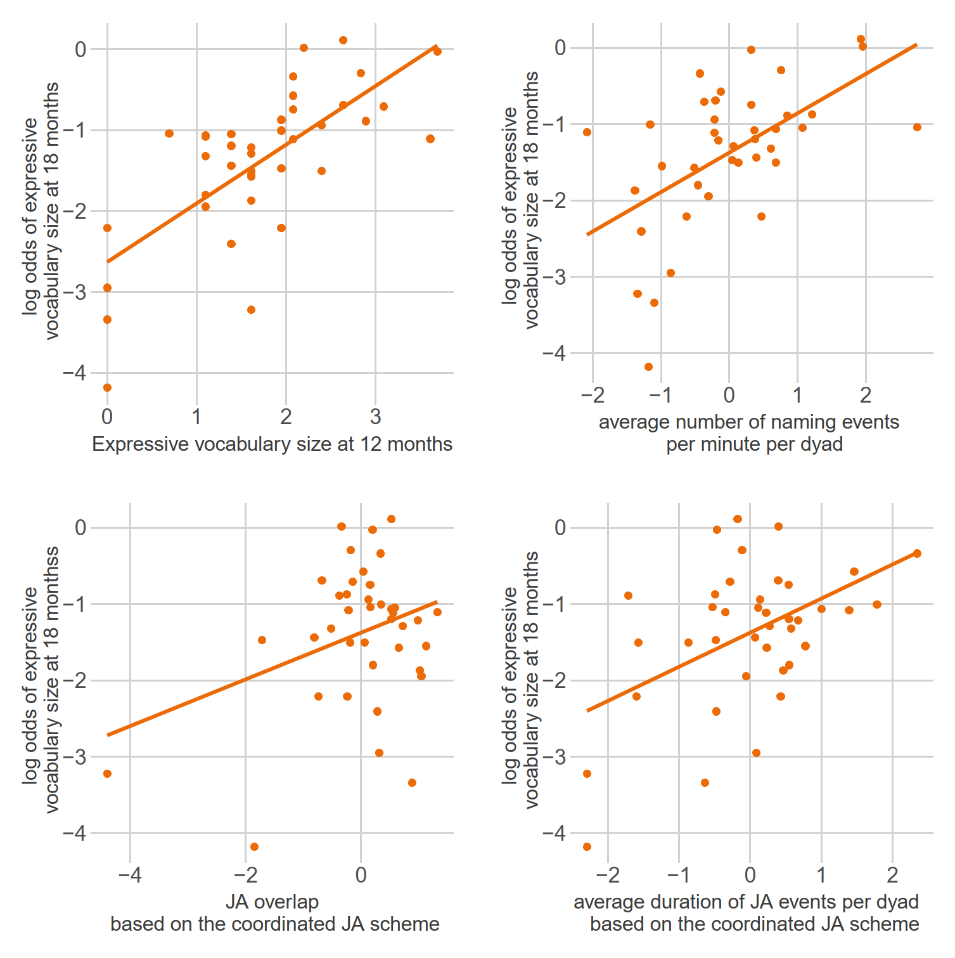
**Supplementary** **Figure 3***Log odds of a vocabulary item of the expressive CDI at 18 months being reported as known and used, modelled as a function of the Predictors of the Coordinated JA Scheme Model*

*Note.* Log odds of a vocabulary item of the expressive CDI at 18 months being reported as known and used (y-axis) modelled as a function of the observed values (x-axis) for the log-transformed predictors based on the *coordinated JA scheme*: the two baseline measures in the upper row*: expressive vocabulary size at 12 months* (top left), and the *average number of naming events per minute* (top right); and the two coding scheme specific measures on the bottom: *JA overlap* (left), and *average JA event duration* (right) stemming from the *coordinated JA scheme*. Lines show regression line, dots show scores for individual dyads.

**Comparison of the coding scheme specific models**

In this section, we use model comparison to evaluate the performance of our two joint attention models. First, we recapitulate the AIC and BIC values of both models from our previous analyses. Second, we report on the results of a 10-fold cross-validation for robust model performance estimation.

The *coordinated JA scheme* model yielded both a lower AIC and a lower BIC, suggesting a better fit to the vocabulary data than the *gaze overlap scheme* model. See Supplementary Table 2 for the results.

**Supplementary Table 2**

*AIC and BIC values of both models, the gaze overlap scheme model and the coordinated JA scheme model*

|  | **Gaze overlap model** | **Coordinated JA model** | **Δ** |
| --- | --- | --- | --- |
| **AIC** | 2789.29 | 2508.13 | **ΔAIC: 281.16** |
| **BIC** | 2797.61 | 2516.45 | **ΔBIC: 281.16** |

For the 10-fold cross validation, the dataset was divided into ten equal parts of which nine folds were used for training and the remaining fold was used for testing. Testing was repeated ten times, each time with a different fold being the test set. The evaluation was based on three metrics: the mean absolute error (MAE), the root mean squared error (RMSE), and R^2^. MAE and RMSE both quantify prediction error, with lower values indicating a better model fit. R^2^ indicates the proportion of variance in the dependent variable that is captured by the model, with higher values indicating better model fit.

The model based on the joint attention measures from the *coordinated JA scheme* outperformed the model based on the joint attention measures of the *gaze overlap scheme*, evident by the lower mean MAE, lower mean RMSE. However, the *gaze overlap scheme* resulted in a and higher mean R^2^ (see Supplementary Table 3). Based on the cross-validation, the *coordinated JA scheme* model outperforms the *gaze overlap scheme* model.

**Supplementary Table 3**

*Results of the cross validation*

| **Metric** | **Minimum** | **1^st^ Quantile** | **Median** | **Mean** | **3^rd^ Quantile** | **Maximum** | **NA’s** |
| --- | --- | --- | --- | --- | --- | --- | --- |
| **MAE** | | | | | | | |
| Gaze Overlap Model | 0.03 | 0.11 | 0.15 | 0.15 | 0.18 | 0.23 | 0 |
| Coordinated JA Model | 0.07 | 0.12 | 0.13 | 0.14 | 0.16 | 0.19 | 0 |
| **RMSE** | | | | | | | |
| Gaze Overlap Model | 0.04 | 0.12 | 0.16 | 0.18 | 0.23 | 0.32 | 0 |
| Coordinated JA Model | 0.08 | 0.13 | 0.16 | 0.17 | 0.22 | 0.28 | 0 |
| **R²** | | | | | | | |
| Gaze Overlap Model | 0.001 | 0.15 | 0.56 | 0.55 | 0.93 | 0.99 | 0 |
| Coordinated JA Model | 0.002 | 0.32 | 0.43 | 0.51 | 0.83 | 0.86 | 0 |

*Note*. Comparison of MAE, RMSE, and R^2^ for both models, the model based on the *gaze overlap scheme* and the model based on the *coordinated JA scheme*.

**Comparison of JA measures**

Finally, we investigated which measure based on which coding scheme best predicts later vocabulary size. In all analyses above we used two measures: a measure of overlap of JA and naming events (*JA overlap*) and a duration measure of JA (*average duration of JA events*). Our analyses above showed that these measures yielded different result when applied to different coding schemes. When operationalised according to the *gaze overlap scheme*, the *JA overlap* measure predicted vocabulary size, but the *average JA event duration* measure did not. When operationalised according to the *coordinated JA scheme*, both the *JA overlap* as well as the *average JA event duration* predicted vocabulary size.

To gain insight on how predictors are related to each other and to ensure that our models are not built with redundant information, we first assessed the correlations between our measures (see main Manuscript). We then conducted a model averaging procedure. Model averaging is useful for cases where competing models could explain the data in different ways. By averaging over multiple models rather than relying on a single “best” model, model averaging procedures allow us to evaluate how different predictors perform across multiple models. This enables us to better understand the relative contribution of the coding-scheme specific measures to predicting vocabulary.

Model averaging was conducted using the MuMIn package in R, identifying the predictors out of all available predictors that are consistently important for explaining the dependent variable. We included both baseline predictors, the *number of naming events per minute* and the concurrent *expressive vocabulary size at 12 months*, and the two coding scheme-specific predictors from both coding schemes, *JA overlap* and *average JA event duration*. The dependent variable was again the *expressive vocabulary size at 18 months*. The full average model was identified by averaging all candidate models whose ΔAIC with the best model was below 5, accounting for model uncertainty.

The results indicated that the two baseline predictors, the *number of naming events per minute* and the concurrent *expressive vocabulary size at 12 months*, showed again a significant positive effect on the proportion of CDI items known and produced at 18 months (*number of naming events per minute*: *β =* 0.44, *z = 19.52*, *p < .001;* expressive *vocabulary at 12 months: β =* 0.81, *z =* 27.19, *p< .001*). For the coding-scheme-specific measures, the *JA overlap* measure as well as the *average JA event duration* from the *gaze overlap scheme* were significant positive predictors of later language (*JA overlap*: *β = 0.08, z = 3.62, p < .001; average JA event duration: β = -0.23, z = 9.16, p < .001*). The *average JA event duration* measure stemming from the coordinated JA scheme was a significant predictor of later language (*average JA event duration:* *β = 0.45, z = 17.92, p < .001*). However, the *JA overlap* measure stemming from the *coordinated JA scheme* was not a predictor of later language (*JA overlap: β = -0.0004, z = 0.027, p = 0.98*). The model-averaged coefficients are summarized in Supplementary Table 4.

**Supplementary Table 4**
Results of the Model Averaging Procedure

|  | **Expressive Vocabulary at 18 months (CDI)** | | | |
| --- | --- | --- | --- | --- |
| *Predictors* | *Estimates* | *SE* | *z* | *p* |
| (Intercept) | -2.79 | 0.06 | 43.59 | <.001*** |
| Expressive vocabulary size at 12 months | 0.81 | 0.03 | 27.19 | <.001*** |
| Number of naming events per minute | 0.44 | 0.02 | 19.52 | <.001*** |
| Coordinated JA scheme: average JA event duration | 0.45 | 0.02 | 17.92 | < .001*** |
| Gaze overlap scheme: average JA event duration | -0.23 | 0.02 | 9.16 | <.001*** |
| Gaze overlap scheme : JA overlap | 0.08 | 0.02 | 3.62 | <.001*** |
| Coordinated JA scheme: JA overlap | -0.0004 | 0.01 | 0.03 | 0.98 |

*p < .05. **p < .01. ***p < .001

Note. Effect on expressive vocabulary size at 18 months of all potential predictors: expressive vocabulary size at 12 months, number of naming events per minute, JA overlap (gaze overlap scheme), average JA event duration (gaze overlap scheme), JA overlap (coordinated JA scheme), average JA event duration (coordinated JA scheme).

**Summary**

Overall, both coding scheme-specific JA models outperform the baseline model, each with significant coding scheme-specific predictors of later expressive vocabulary scores. The model based on the predictors stemming from the *coordinated JA scheme* outperforms the model based on the predictors stemming from the *gaze overlap scheme* (based on AIC and BIC comparison, MAE and RMSE comparison, but note a lower R^2^ in the cross validation). However, differences in the predictor strength can be observed. Nevertheless, the analyses for expressive vocabulary at 18 months supports our general finding of coding-scheme specific variation in the prediction ability of JA measures, and adds a layer of age-dependent sensitivity to it.
